# e-RNA: a collection of web servers for comparative RNA structure prediction and visualisation

**DOI:** 10.1093/nar/gku292

**Published:** 2014-05-07

**Authors:** Daniel Lai, Irmtraud M. Meyer

**Affiliations:** Centre for High-Throughput Biology, Department of Computer Science and Department of Medical Genetics, University of British Columbia, Vancouver V6T 1Z4, Canada

## Abstract

e-RNA offers a free and open-access collection of five published RNA sequence analysis tools, each solving specific problems not readily addressed by other available tools. Given multiple sequence alignments, Transat detects all conserved helices, including those expected in a final structure, but also transient, alternative and pseudo-knotted helices. RNA-Decoder uses unique evolutionary models to detect conserved RNA secondary structure in alignments which may be partly protein-coding. SimulFold simultaneously co-estimates the potentially pseudo-knotted conserved structure, alignment and phylogenetic tree for a set of homologous input sequences. CoFold predicts the minimum-free energy structure for an input sequence while taking the effects of co-transcriptional folding into account, thereby greatly improving the prediction accuracy for long sequences. R-chie is a program to visualise RNA secondary structures as arc diagrams, allowing for easy comparison and analysis of conserved base-pairs and quantitative features. The web site server dispatches user jobs to a cluster, where up to 100 jobs can be processed in parallel. Upon job completion, users can retrieve their results via a bookmarked or emailed link. e-RNA is located at http://www.e-rna.org.

## INTRODUCTION

Recent studies have shown the expanding number of functional RNAs within the cell (1). In many cases, the function of the RNA is conferred by its structure, such as loop binding domains on long non-coding RNAs (2), short catalytic hairpin targets for RNA editing and RNA silencing (3), and self-regulating multi-conformation riboswitches (4). While experimental techniques for the high-throughput determination of RNA secondary structure have seen recent developments (5), their computational prediction also remains a fast and cost-efficient method for researchers to study RNA secondary structures and their potential functional roles (6).

We here focus on the prediction of RNA secondary structure (henceforth referred to simply as structure), i.e. the set of Watson-Crick and wobble base-pairs. We have developed a web server hosting five tools previously published by our group, giving users a user-friendly interface for predicting specific sequence features currently only possible with our tools. Transat (7) is able to detect all conserved helices, including mutually incompatible helices such as those found in some riboswitches. RNA-Decoder (8) was designed to be able to detect conserved structure overlapping protein-coding regions, demonstrated in viral sequences. Given a set of homologous input sequences, SimulFold (9) outputs the conserved structure (including pseudo-knots) as well as the consensus sequence alignment with prior probabilities for each. CoFold (10) predicts the minimum-free energy (MFE) structure while explicitly considering the effects of co-transcriptional folding. Finally, to visualise the results the above algorithms we use R-chie (11), a collection of tools to create arc and covariance plots.

## PROGRAMS

### Transat

There already exist several algorithms that take in a multiple sequence alignment and predict the conserved structure, Pfold (12) and RNAalifold (13) being examples. Transat uses the same evolutionary models as Pfold, but instead of restricting the final solution to a set of compatible helices predicted to be part of a single structure, it instead recovers and scores all conserved helices. Transat is therefore invaluable when aiming to find transient, alternative and pseudo-knotted helices which are ignored by other programs (14).

**Figure 1.**
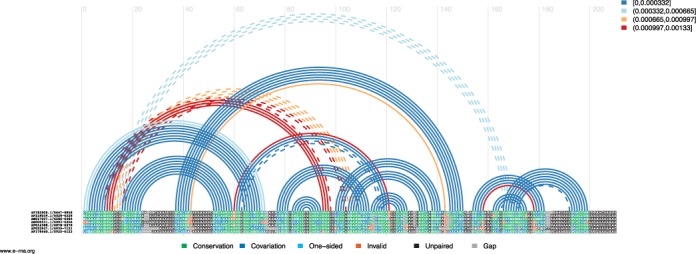
Arc plot visualising the predictions by the Transat web server. Each arc connects two positions that correspond to a base-pair and is coloured according to its estimated *p*-value, see legend in the top right. Solid arcs indicate arcs with mutually exclusive positions, while dashed arcs indicate those that overlap with an existing (and better) base-pair. Below the arcs, a covariance plot is shown, displaying the nucleotide of each sequence at each position. If two columns are base-paired and connected by a solid arc, we colour the two columns according to how conserved the base-pair is, green for fully conserved (no mutations), blue for compensatory mutations, and red for a loss of base-pairing.

The web server interface consists of an input box for the input multiple alignment sequence in FASTA format, an optional input box for a phylogenetic tree in Newick format, and a set of parameter options to adjust, if desired. If no phylogenetic tree is provided, it will be computed internally using programs distributed with Pfold (12). Our server runs the identical algorithm as published previously by (7), but re-implemented to run in a fraction of the previously reported run-time. The output consists of a set of helices, each assigned a *p*-value and other statistics in tab delimited format which allow the user to easily rank the helices. We also provide output plots in PNG and PDF format for the top 25 helices in an arc plot format, where the horizontal line presents the input alignment, and arcs connecting the two positions involved in each base-pair (Figure 1). Underneath the arc plot, we present a covariance plot, showing the location of mutations in the base-paired columns, highlighting any compensatory mutations.

### RNA-Decoder

Whereas tools like Transat and Pfold predict conserved RNA structure by detecting the unique evolutionary pattern according to which paired and unpaired nucleotides mutate, this can be further complicated when the sequences are partly protein-coding, as codons evolve differently. To address structure overlapping protein-coding regions, dedicated evolutionary models were developed (15), implemented in the comparative structure prediction program RNA-Decoder (8). RNA-Decoder has since been successfully used to detect conserved structures in human pre-mRNAs (16) as well as entire viral genomes (17). As of now it is still the only comparative structure prediction program that takes known protein-coding regions explicitly into account.

**Figure 2.**
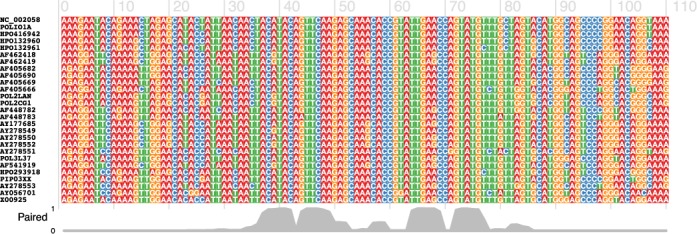
Pairing probability figure directly output from the RNA-Decoder web server, showing the probability of a single viral hairpin structure.

The RNA-Decoder web server takes as input a multiple sequence alignment in FASTA format, with an optional annotation line consisting of a sequence of the numbers 1, 2 or 3 to denote the protein-coding positions and 3 to denote any non-coding positions. If no codon annotation is specified, an automated attempt is made to recover the codons by translating the sequence. As optional input, a phylogenetic tree can be provided. RNA-Decoder can then be used in one of two modes, fold or scan. In folding mode, RNA-Decoder returns the conserved RNA structure (without pseudo-knots) with corresponding prior probabilities for the predicted annotation state of each alignment column (i.e. paired, unpaired, or unstructured). In scanning mode, RNA-Decoder returns base-pairing probabilities for each *individual* alignment column rather than a structure with specific base-pairs (Figure 2).

The program run on the server is the original unaltered program released by (8). Similar to the original manuscript, input alignments are split into sliding windows of 600 bps width, each overlapping by 400 bps to efficiently process large inputs in both modes. When visualised, overlapping regions take the maximum pairing probability in scanning mode, and the most likely structure and state when folding.

### SimulFold

All programs that detect conserved RNA structure based on a fixed multiple sequence input alignment require a high-quality input alignment to reliably detect the conserved structure. Often, this implies that a alignment of high quality cannot be compiled unless the conserved structure is known upfront, amounting to a serious chicken-egg problem. A few existing tools tackle this problem by simultaneously aligning and folding a given multiple sequence alignment, such as Foldalign (18), carnac (19) and carna (20). These tools however, are either limited to two input sequences only, are unable to model pseudo-knots, or fail to model the phylogenetic relationship between sequences. SimulFold which employs a Bayesian Markov chain Monte Carlo was especially developed to overcome these problems and is capable of simultaneously co-estimating the conserved RNA structure (including pseudo-knots), the multiple sequence alignment and the phylogenetic tree.

The SimulFold web server takes a set of homologous sequences in the form of a FASTA format multiple sequence alignment and an optional phylogenetic tree in Newick format. The initial alignment is subsequently used as the starting point for the Markov chain Monte Carlo and may thus correspond to a sub-optimal alignment generated by one of the programs that only capture primary sequence conservation. The user then gets to choose three sampling options for structure, alignment, and tree, that when selected, allow the algorithm to sample the respective feature. Users can thus choose to only co-estimate the structure and alignment if, say, a trustworthy phylogenetic tree is known upfront. The web server uses the original unmodified program as published in (9). The output is shown as an arc plot and covariance plot made using R-chie, along with a figure of the alignment showing the prior probability of aligning the nucleotides in each column.

### CoFold

When trying to predict the RNA structure for a single input sequence, the most common strategy is to determine the (pseudo-knot-free) RNA structure that minimises the overall free energy of the molecule. This MFE structure, however, does not account for the effects of co-transcriptional folding that may influence structure formation *in vivo* (21). By creating the non-comparative structure prediction program CoFold (10), we have shown that explicitly capturing some effects of co-transcriptional folding in an MFE prediction program can greatly increase the prediction accuracy, especially for sequences longer than 1000 nt.

The web interface of CoFold takes as input a single sequence in FASTA format. The user can optionally choose one of two commonly used energy parameters (22, 23) and can alter the two scaling parameters of CoFold, if desired, which alter the impact of co-transcriptional folding on structure formation. The underlying algorithm is the one described in the original manuscript (10). The resulting output is a single, pseudo-knot-free RNA secondary structure.

### R-chie

Finally, we provide a web server for the visualisation tool R-chie (11). This tool allows the visualisation of numerous RNA secondary structure features as arc diagrams. One key feature is the optional, simultaneous visualisation of corresponding multiple sequence alignments and their degree of structure-related conservation, in particular compensatory mutations within base-pairs. In contrast to other visualisation programs, arc diagrams generated by R-chie can also show conflicting base-pairs (dashed lines in Figure 1), and allow the ready comparison of multiple structures against the same multiple-sequence alignment (7). Due to the RNA sequence or multiple sequence alignment being represented as a horizontal line in arc diagrams, it is easy to annotate structure motifs (24), and to visualise quantitative scores assigned to base-pairs or individual sequence positions, e.g. the result of chemical probing (25).

In addition to arc diagrams, R-chie can create covariance plots, which are a combination of the aforementioned arc diagrams with a corresponding, coloured multiple sequence alignment underneath (Figure 1 is one such example). While it is possible to summarise the degree of conservation for each base-pair using a numerical score such as percent identity or covariance, a covariance plots generated by R-chie readily displays for each sequence and position the type of mutation or conservation. Rfam (26) now uses R-chie diagrams to visualise RNA structure features in conjunction with the underlying alignments.

The most basic usage of R-chie requires only an input RNA secondary structure in one of several commonly used formats, including dot-bracket and connect format. Supplying two structures as input for the same sequence or multiple sequence alignment allows the simultaneous visualisation of the two structures along the same horizontal line. Finally, the addition of a multiple sequence alignment as input, triggers the additional display of the corresponding covariance plot for the provided structure(s). For the full description of all input and other options, please refer to the original manuscript (11). R-chie returns figures in either PNG or PDF format, and also specifies the corresponding command line that users could use to reproduce the same figure with their own copy of R-chie.

## CONCLUSION

e-RNA provides a collection of unique programs for RNA secondary structure prediction and visualisation, with an emphasis on the detection of conserved RNA structure features. Using a multi-core computer cluster and a job scheduling system, our servers can process up to 100 jobs in parallel. Upon job completion, users can either retrieve their results via a job-specific bookmarked link, or optionally receive the link via email, if the email address was supplied during job submission. For most of our tools, a tar-ball file containing the full job input, intermediate files, output and figures is provided for download for further analysis by the user. We provide this server in the hope that it will be useful, but it is provided as is without any warranty of any kind, expressed or implied. Finally, as research continues in our lab, we will continue to make additions and updates to e-RNA.
